# Bardoxolone Methyl Prevents Fat Deposition and Inflammation in Brown Adipose Tissue and Enhances Sympathetic Activity in Mice Fed a High-Fat Diet

**DOI:** 10.3390/nu7064705

**Published:** 2015-06-09

**Authors:** Chi H. L. Dinh, Alexander Szabo, Yinghua Yu, Danielle Camer, Qingsheng Zhang, Hongqin Wang, Xu-Feng Huang

**Affiliations:** 1 Centre for Translational Neuroscience, School of Medicine, University of Wollongong and Illawarra Health and Medical Research Institute, Wollongong, NSW 2522, Australia; E-Mails: hlcd893@uowmail.edu.au (C.H.L.D.); a.m.szabo@gmail.com (A.S.); yinghua@uow.edu.au (Y.Y.); dc608@uowmail.edu.au (D.C.); kiefer@uow.edu.au (Q.Z.); hongqin@uow.edu.au (H.W.); 2 ANSTO LifeSciences, Australian Nuclear Science and Technology Organisation, Lucas Heights, NSW 2234, Australia

**Keywords:** obesity, brown adipose tissue, brainstem, bardoxolone methyl, high-fat diet

## Abstract

Obesity results in changes in brown adipose tissue (BAT) morphology, leading to fat deposition, inflammation, and alterations in sympathetic nerve activity. Bardoxolone methyl (BARD) has been extensively studied for the treatment of chronic diseases. We present for the first time the effects of oral BARD treatment on BAT morphology and associated changes in the brainstem. Three groups (*n =* 7) of C57BL/6J mice were fed either a high-fat diet (HFD), a high-fat diet supplemented with BARD (HFD/BARD), or a low-fat diet (LFD) for 21 weeks. BARD was administered daily in drinking water. Interscapular BAT, and ventrolateral medulla (VLM) and dorsal vagal complex (DVC) in the brainstem, were collected for analysis by histology, immunohistochemistry and Western blot. BARD prevented fat deposition in BAT, demonstrated by the decreased accumulation of lipid droplets. When administered BARD, HFD mice had lower numbers of F4/80 and CD11c macrophages in the BAT with an increased proportion of CD206 macrophages, suggesting an anti-inflammatory effect. BARD increased phosphorylation of tyrosine hydroxylase in BAT and VLM. In the VLM, BARD increased energy expenditure proteins, including beta 3-adrenergic receptor (β_3_-AR) and peroxisome proliferator-activated receptor gamma coactivator 1-alpha (PGC-1α). Overall, oral BARD prevented fat deposition and inflammation in BAT, and stimulated sympathetic nerve activity.

## 1. Introduction

Obesity is associated with serious health effects, such as type 2 diabetes and heart disease. Obesity affects the health of various tissues in the periphery (*i.e.*, white and brown adipose tissue) and the brain [1,2,3]. Brown adipose tissue (BAT) is the primary site for non-shivering thermogenesis and energy expenditure. In contrast to white adipose tissue (WAT) which stores energy, BAT dissipates energy from food as heat, leading to a reduction in fat storage and weight gain [4]. BAT is present in human adults [5,6], and hence has been extensively investigated as a therapeutic target to prevent and treat obesity.

BAT is higly innervated by sympathetic nerves and the brainstem is an autonomic center for sympathetic outflow to this tissue [7]. The noradrenergic nucleus in the brainstem contains sympathetic nerves which releases norepinephrine and innervates tissues, including BAT. The rate-limiting enzyme tyrosine hydroxylase (TH) is a marker of noradrenergic nerve fibers [8]. TH immunoreactivity in BAT is lower in obesity-prone C57BL/6J mice than in obesity-resistant Sv129 mice [9]. A reduced level of TH mRNA has also been found in the brainstem of olanzapine-induced overweight rats [10].

Adrenergic activation contributes to energy regulation in BAT and the brain. Beta 3-adrenergic receptor (β_3_-AR), peroxisome proliferator-activated receptor gamma coactivator 1-alpha (PGC-1α) and uncoupling proteins (UCPs) have been found in BAT and in many brain regions, and are involved in energy regulation [11,12,13,14,15]. Reduced levels of these proteins have been found in the WAT and BAT of obese mouse models [16,17,18]. However, the expression of these proteins in BAT and brainstems of HFD-fed mice during dietary intervention has not been examined.

Obesity is associated with morphological changes in BAT, and in particular functional BAT is reduced in obese and diabetic patients [19]. Moreover, excess fat deposition in BAT has been observed in obese rodent models [20,21,22,23]. Conversion of BAT into WAT-like tissue has been demonstrated in obese prone mice [17]. Other studies have shown that BAT of mice fed high-energy diets (HFD and cafeteria diet) is highly infiltrated with macrophages [3], and macrophages are recruited as part of the inflammatory phenotype in BAT of diabetic prone mice [24].

Pentacyclin triterpenes are chemical compounds extracted from herbal medicines, which can be found in plant roots, seeds, leaves and fruits (e.g., ginseng, tea and apples). These compounds act on multiple tissues, including BAT and brain [25,26]. Among these triterpenes, oleanolic acid and its derivatives demonstrate myriad benefits in the defence against inflammation, type 2 diabetes and associated disorders [27,28]. Synthetic oleanane triterpenoids have potent therapeutic properties for the prevention and treatment of chronic diseases [29,30]. Bardoxolone methyl (BARD), a C-28 methyl ester of 2-cyano-3, 12-dioxoolean-1,9-dien-28-oic acid (CDDO), is one of these synthetic oleanolic acids. Human and animal studies have illustrated anti-obesity, anti-diabetic, and anti-inflammatory actions of BARD [31,32,33,34]. BARD is absorbed through small intestine mucosa and distributes to distal tissues such as cerebral cortex and lung [35]. It targets immune cells in multiple organs including brain, kidney, and WAT [27,31,36,37]. BARD increases energy expenditure in HFD mice by elevating oxygen consumption [31]. Additionally, we have found that BARD increases uncoupling and other energy expenditure proteins in WAT of HFD-fed mice [16]. However, the effects of BARD on BAT morphology and the sympathetic nervous system have not been studied yet, and its role in energy regulation is not clear. 

In this study, we investigated the effect of BARD on BAT morphology and molecular changes in HFD-fed mice. We also assessed the effects of BARD in the ventrolateral medulla (VLM) and dorsal vagal complex (DVC) of the brainstem. Current study has shown that BARD is well tolerated and effective in obese and diabetic rodents [38]. Present study may not only help to understand BARD pharmacology in the BAT and brainstem axis, but also to investigate the potential of this compound in the prevention of obesity associated complications.

## 2. Materials and Methods

### 2.1. Animals

Twenty one C57BL/6J male mice were obtained from the Animal Resource Centre (Perth, Australia), and acclimatized within our institutional animal facility (temperature 22 °C, 12 h light/dark cycle) for one week before experimentation. All procedures were approved by the Animal Ethics Committee, University of Wollongong, NSW, Australia, and complied with the *Australian Code of Practice for the Care and Use of Animals for Scientific Purposes.*

Animals were divided into three groups (*n =* 7), and fed either a high-fat diet (HFD), a high-fat diet supplemented with bardoxolone methyl (HFD/BARD), or a low-fat diet (LFD). The HFD and HFD/BARD groups were fed a HFD containing 40% energy from fat (SF11-095, Specialty Feeds, WA, USA), and the low-fat diet (LFD) animals were maintained on normal diet (Vella Stock Feeds, Doonside, NSW, Australia). The dose of BARD was selected as 10 mg/kg body weight, according to dosages from previous studies [31,33], and was administered in drinking water for 21 weeks. Body weight was measured before and after the experiment, which shows significant reduction of body weight in HFD mice administered BARD [16]. HFD and LFD control animals received saline in drinking water. Samples of interscapular BAT were fixed in 4% paraformaldehyde and embedded in paraffin for histology and immunohistochemistry. Other samples of interscapular BAT and brain were snap-frozen in liquid N_2_ and stored at −80 °C for Western blot. 

Frozen brain sections were cut at 400 µm based on a standard mouse brain atlas according to our previous study [39,40]. Brainstem VLM and DVC were collected from sections using a Stoelting Brain Punch (#57401, 0.5 mm diameter, Wood Dale, Stoelting Co., IL, USA). The brainstem samples were stored at −80 °C for further analysis. 

### 2.2. Histological and Immunohistochemical Staining

For histology, paraffin embedded BAT was sectioned at 4 µm and stained with haematoxylin and eosin (POCD Scientific, Artarmon, NSW, Australia). Microphotographs were taken using a Leica microscope (×40). ImageJ 1.46r software (National Institute of Health, Bethesda*,* MD, USA) (http://imagej.nih.gov/ij/download.html) was used to quantify the size of lipid area and lipid droplets [22,41,42]. Three fields per section and three sections per fat mass were used for statistical analysis. 

Immunohistochemical staining was used to assess the density of the total macrophages, inflammatory macrophage phenotype (M1) and anti-inflammatory macrophage phenotype (M2). All antibodies were purchased from Abcam Inc, Cambridge, MA, USA. Antigen retrieval was performed by microwaving paraffin embedded sections of BAT (4 µm) in sodium citrate buffer (10 mM, pH 6.0). The sections were then washed in 0.3% H_2_O_2_ in methanol for 10 min, blocked with 5% normal rabbit serum, and incubated overnight at 4 °C with primary antibodies. Primary antibodies were anti-F4/80 (ab6640), anti-CD11c (ab33483) and anti-CD206 (ab64693). Sections were then incubated consecutively with the appropriate secondary anti-bodies: rabbit anti-rat IgG biotin (ab6733), goat anti-armenian hamster IgG H&L biotin (ab5744), goat anti-rabbit IgG H&L biotin (ab6720). The sections were then incubated with streptavidin-HRP polymer conjugate (#2438, Sigma-Aldrich Pty. Ltd, Sydney, NSW, Australia) for 30 min at room temperature. Samples were then developed using the ImmPACT DAB peroxidase substrate kit (#4100, Vector laboratories Inc., Burlingame, CA, USA) and counterstained with haematoxylin (POCD Scientific, Artarmon, NSW, Australia). Microphotographs were taken using a Leica microscope (×40). Three fields per section and three sections per fat mass were used for data analysis. ImageJ 1.46r software was used for the quantification of macrophages.

### 2.3. Western Blot Analysis

Western blot was used to quantify changes in the expression of energy expenditure proteins. This procedure was performed as previously described [43]. Briefly, the protein concentration of BAT NP-40 lysis buffer extracts was determined using the Thermo Scientific Pierce™ BCA Protein Assay Kit (Pierce Chemical Co., Rockford, IL, USA). Protein within the lysates (25 μg) was then separated on Bio-Rad 4%–12% Bis Tris-HCl gels, 26 wells (Bio-rad laboratories, Gladesville, Australia), and transferred to a polyvinylidene difluoride (PVDF) membrane. The antibodies used to identify protein expression were anti-tyrosine hydroxylase phosphoSer 40 (pTH) (AB5935), anti-tyrosine hydroxylase (TH) (AB9983), and anti-actin (β-actin) (MAB1501) from Merck Millipore (Kilsyth, VIC, Australia); anti-UCP1 (sc-6529), anti-β_3_-AR (sc-1473), anti-PGC-1α (sc-13067), anti-UCP2 (sc-6525) from Santa Cruz Biotechnology (Dallas, TX, USA). 

After overnight incubation with the primary antibodies, the samples were incubated for 1 h in the appropriate horseradish peroxidase conjugated secondary antibodies, goat anti-rabbit (AP307P) and goat anti-mouse (AP308P) from Chemicon International Inc (Temecula, CA, USA); and donkey-anti-goat (sc-2033) from Santa Cruz Biotechnology (Dallas, TX, USA). The protein targets were then detected using enhanced chemiluminescence buffer from GE Healthcare, (Piscataway, NJ, USA). Quantity One software (Bio-Rad Laboratories, Hercules, CA, USA) was used to quantify the protein bands based on the ratio between the band for the protein of interest and β-actin. 

### 2.4. Statistical Analysis

We used the SPSS 19 package (SPSS, Chicago, IL, USA) for data analysis. All data are presented as mean ± standard error of the mean (SEM). One way analysis of variance (ANOVA) and the least significant difference (LSD) post-hoc analysis were used to compare the morphology of adipocytes (lipid area and lipid droplet area), density of macrophages, and the expression of sympathetic proteins among mouse groups (LFD group, HFD group, and HFD/BARD group). Differences between groups were considered statistically significant at *p <* 0.05.

## 3. Results 

### 3.1. BARD Prevents Fat Deposition in the BAT of Mice Fed a HFD

**Figure 1:**
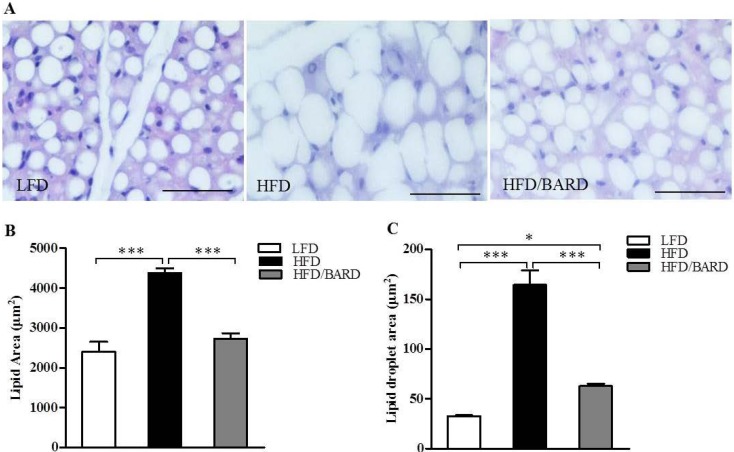

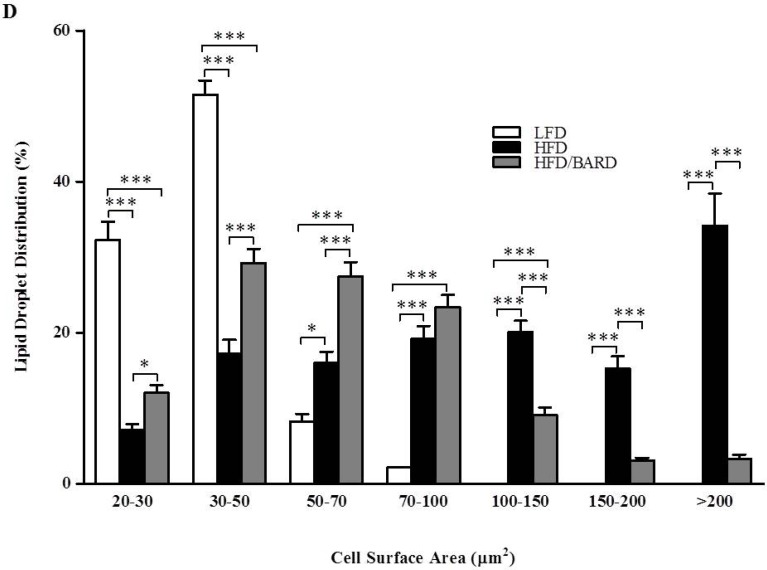
Effect of bardoxolone methyl (BARD) on fat deposition in brown adipose tissue of mice fed: low-fat diet (LFD), high-fat diet (HFD), and high-fat diet supplemented with BARD (HFD/BARD). (**A**) Haematoxylin and eosin staining of brown fat pads (×40). Bar = 50 µm; (**B**) Lipid area; (**C**) Lipid droplet area; (**D**) Distribution of lipid droplets. All data are presented as mean ± SEM. * *p <* 0.05, *** *p <* 0.001.

In histological micrographs, HFD mice had larger lipid droplets than LFD and HFD/BARD mice (Figure 1A). HFD mice had an 82% increase in lipid area compared with the LFD mice (*p <* 0.001) (Figure 1B). Supplementing the diet with BARD reduced the lipid area by 38% compared to HFD mice (*p <* 0.001). Compared to the LFD mice, HFD mice had a five-fold increase in lipid droplet diameter (*p <* 0.001) (Figure 1C). In contrast the diameter of lipid droplets decreased three-fold when HFD mice were administered BARD (*p <* 0.001). Additionally, HFD/BARD mice had larger numbers of small lipid droplets compared with HFD mice (Figure 1D). This data indicated a preventive effect of BARD on HFD-induced fat deposition in BAT. 

### 3.2. BARD Prevents Macrophage Infiltration and Recruitment of CD11c and CD206 in BAT of Mice Fed a HFD

**Figure 2:**
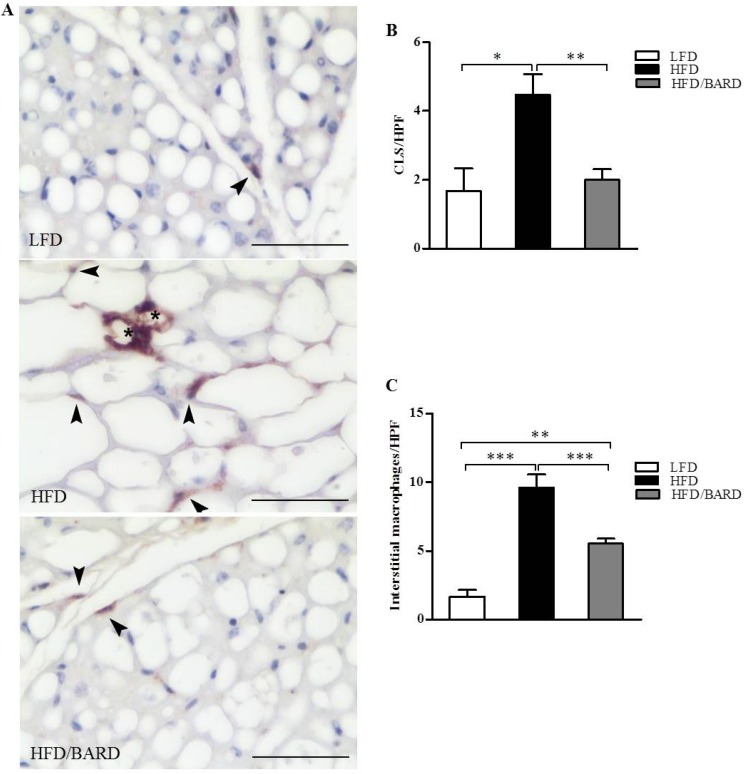
Effect of bardoxolone methyl (BARD) on the expression of F4/80 macrophages in brown adipose tissue of mice fed: low-fat diet (LFD), high-fat diet (HFD), and high-fat diet supplemented with BARD (HFD/BARD). (**A**) F4/80 stained sections (×40). Bar = 50 µm; (**B**) Number of crown-like structures (CLS) per high-power field (HPF); (**C**) Number of interstitial macrophages per HPF. The asterisks illustrate CLS while the arrowheads demonstrate single interstitial macrophages. All data are presented as mean ± SEM. * *p <* 0.05, ** *p <* 0.01, *** *p <* 0.001.

Immunohistochemistry was used to examine the effect of BARD on the density of macrophages in BAT (Figure 2A). The data show that there was a significant increase in the number of F4/80-positive crown-like structures, by 168%, in HFD compared with LFD mice (*p <* 0.05). In contrast, the number of crown-like structures was significantly reduced by 55% when HFD mice were administered BARD (*p <* 0.01) (Figure 2B). In Figure 2C, the number of interstitial macrophages in HFD mice was significantly increased compared with LFD mice (+48%, *p <* 0.001) and mice administered HFD supplemented with BARD (42%, *p <* 0.001). 

**Figure 3:**
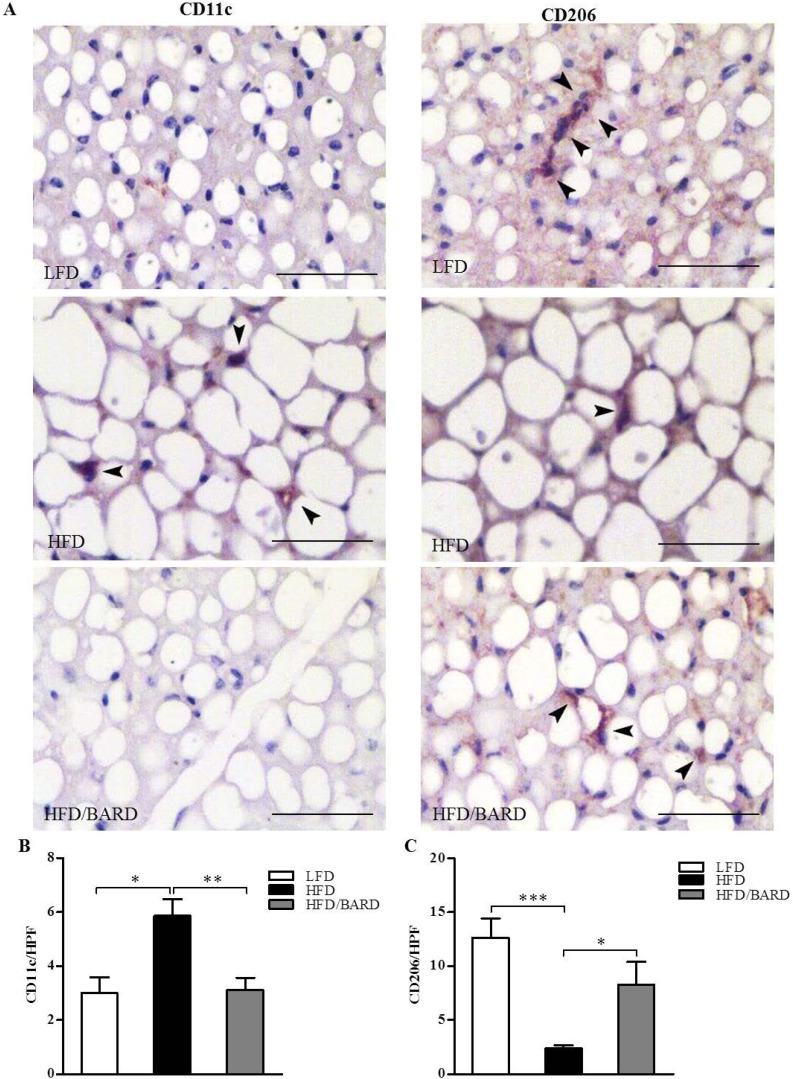
Effects of bardoxolone methyl (BARD) on the expression of CD11c and CD206 macrophages in brown adipose tissue of mice fed: low-fat diet (LFD), high-fat diet (HFD), and high-fat diet supplemented with BARD (HFD/BARD). (**A**) CD11c and CD206 stained sections (×40). Bar = 50 µm; (**B**) Number of CD11c-positive stained cells per high-power field (HPF); (**C**) Number of CD206-positive stained cells per HPF. All data are mean ± SEM. * *p <* 0.0.05, ** *p <* 0.01, *** *p <* 0.001.

We investigated the distribution of inflammatory (M1) and anti-inflammatory (M2) macrophage phenotypes in BAT by immunohistochemical staining for CD11c and CD206, respectively (Figure 3A). Compared with LFD mice, HFD mice had a significant increase in the number of CD11c positive macrophages (+95%, *p <* 0.05) (Figure 3B). BARD administration resulted in a 47% decrease in the number of CD11c positive cells (*p <* 0.01) in HFD mice. On the other hand, HFD mice had 81% fewer CD206 positive macrophages compared with LFD mice (*p <* 0.001) (Figure 3C). While compared with HFD controls, BARD treated mice had greater number of CD206 positive cells (+49%, *p < 0*.01). Taken together, these results suggest the potential of BARD in preventing inflammation in BAT. 

### 3.3. BARD Enhances Noradrenergic Innervation in BAT of Mice Fed a HFD

**Figure 4:**
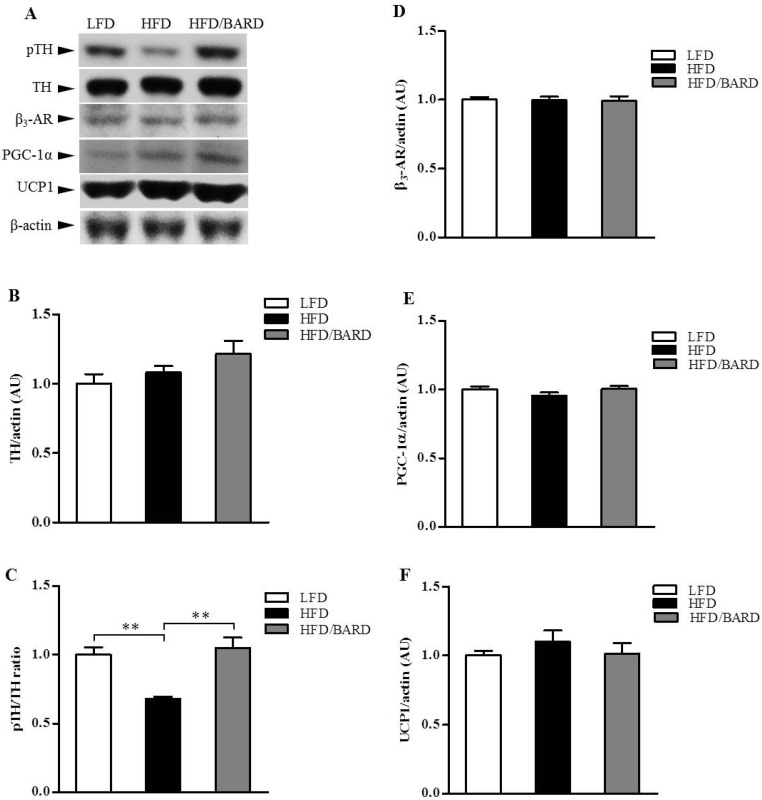
Effect of bardoxolone methyl (BARD) on noradrenergic innervation and energy expenditure proteins in brown adipose tissue of mice fed: low-fat diet (LFD), high-fat diet (HFD), and high-fat diet supplemented with BARD (HFD/BARD). (**A**) Representative blots; (**B**) Total TH protein; (**C**) pTH/TH ratio; (**D**) β_3_-AR protein; (**E**) PGC1-α protein; (**F**) UCP1 protein. All data are mean ± SEM. ** *p <* 0.01. TH: tyrosine hydroxylase; pTH: phosphorylated tyrosine hydroxylase; β_3_-AR: beta 3-adrenergic receptor; PGC1-α: peroxisome proliferator-activated receptor gamma coactivator 1-alpha; UCP1: uncoupling protein 1; AU: arbitrary unit.

To investigate the effects of BARD on sympathetic innervation in BAT, we examined the expression of TH protein and its phosphorylation. The expression of energy expenditure proteins (UCP1, β_3_-AR and PGC-1α) was also determined by Western blot (Figure 4A). As shown in Figure 4C, HFD mice had significant reduction in the phosphorylated (pTH)/TH ratio compared with LFD mice (−32%, *p <* 0.01). BARD administration in HFD mice increased the pTH/TH ratio by 55% (*p <* 0.01) compared with mice fed HFD alone. There was no significant difference in the expression of total TH, β_3_-AR and PGC-1α and UCP1 protein among the three groups of mice (Figure 4B,D–F, respectively). The increased TH signalling activity suggests that BARD activates BAT via noradrenergic innervation.

### 3.4. BARD Enhances Tyrosine Phosphorylation and Energy Expenditure Proteins in Brainstems of Mice Fed a HFD

**Figure 5:**
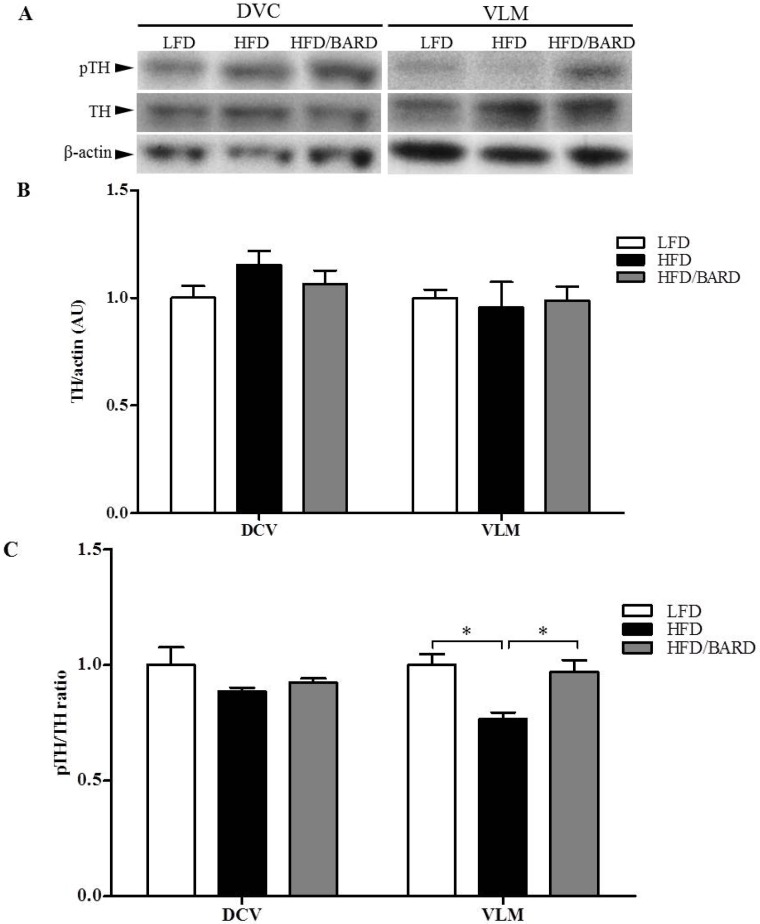
Effect of bardoxolone methyl (BARD) on noradrenergic activation in the brainstem of mice fed: low-fat diet (LFD), high-fat diet (HFD), and high-fat diet supplemented with BARD (HFD/BARD). (**A**) Representative blots; (**B**) Total TH protein; (**C**) pTH/TH ratio. All data are mean ± SEM. * *p* < 0.05. TH: tyrosine hydroxylase; pTH: phosphorylated tyrosine hydroxylase; DVC: dorsal vagal complex; VLM: ventrolateral medulla; AU: arbitrary unit.

We investigated sympathetic activity from the brainstem by assessing the expression of TH signalling in DVC and VLM of the brainstem (Figure 5A). Although total TH protein did not change, alterations in TH signalling were observed in the VLM region of the brainstem (Figure 5B,C, respectively). Compared with LFD mice, the ratio of pTH/TH in HFD mice was reduced by 23% (*p <* 0.05). BARD administration in HFD mice significantly increased pTH/TH ratio by 26% compared with HFD controls (*p <* 0.05). There was no significant difference in expression of total and phosphorylated TH in the DVC region of the brainstem among the three groups of mice. These results suggest that BARD stimulates sympathetic nerves from the VLM region of the brainstem via TH signalling.

**Figure 6:**
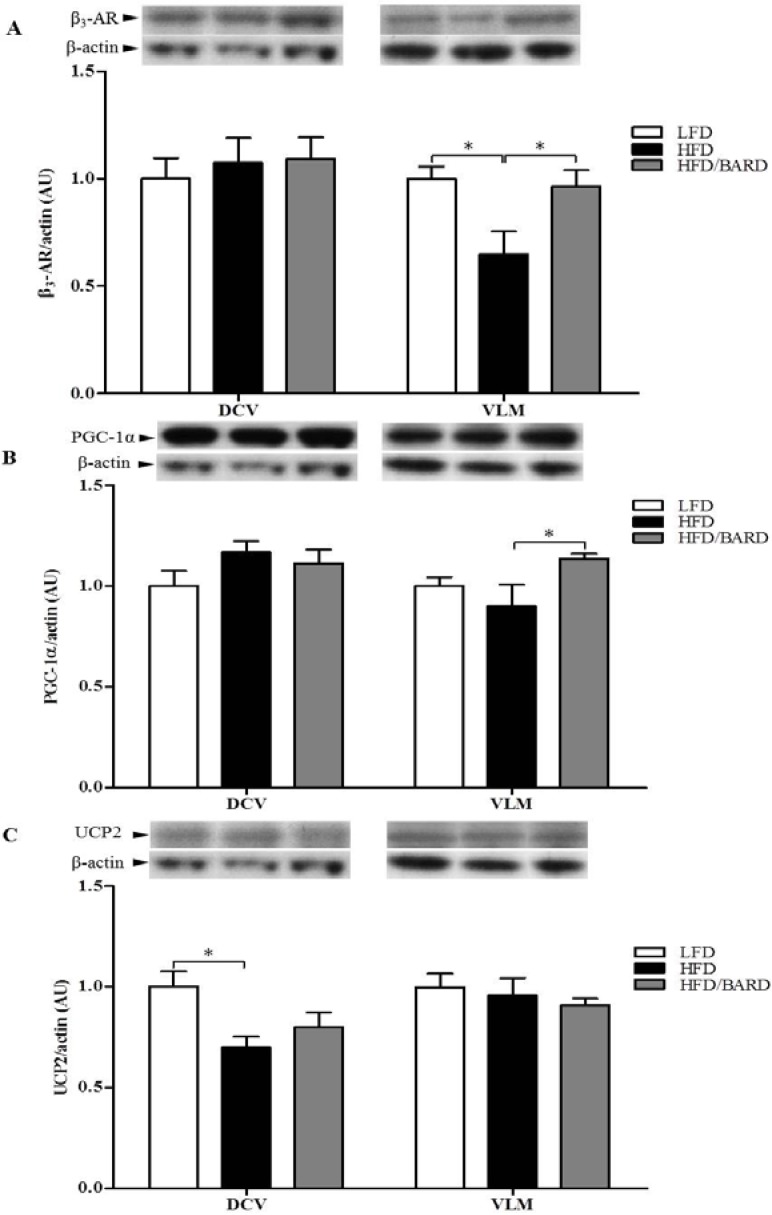
Effect of bardoxolone methyl (BARD) on the expression of energy expenditure proteins in the brainstem of mice fed: low-fat diet (LFD), high-fat diet (HFD), and high-fat diet supplemented with BARD (HFD/BARD). (**A**) β_3_-AR protein and representative blots (**B**) PGC1-α protein and representative blots; (**C**) UCP2 protein and representative blots. All data are expressed as mean ± SEM, * *p <* 0.05, ** *p <* 0.01. β_3_-AR: beta 3-adrenergic receptor; PGC1-α: peroxisome proliferator-activated receptor gamma coactivator 1-alpha; UCP2: uncoupling protein 2; DVC: dorsal vagal complex; VLM: ventrolateral medulla; AU: arbitrary unit.

We further assessed the expression of energy expenditure proteins (β_3_-AR, PGC-1α, UCP2) in the DVC and VLM of the brainstem. In the VLM, HFD mice had reduced protein levels of β_3_-AR (−35%, *p <* 0.05) and PGC-1α (−10%, *p* = 0.25) compared with LFD mice (Figure 6A,B, respectively). In contrast, BARD administration to HFD mice significantly increased protein expression of β_3_-AR by 48% (*p <* 0.01) and PGC-1α by 27% (*p <* 0.05). In the DVC, although UCP2 protein was significantly decreased in HFD mice compared with LFD mice (−30%, *p <* 0.05), BARD did not significantly increase the expression of this protein (Figure 6C).

## 4. Discussion and Conclusions

Studies have demonstrated the preventative effects of BARD on obesity and obesity-associated complications [31,34]. We further show that daily supplementation of BARD in drinking water during HFD feeding prevented fat deposition and inflammation in the BAT of obesity-prone C57BL/6J mice. Additionally, we found that BARD activates sympathetic nerves in BAT and the brainstem and enhanced the expression of energy expenditure proteins in the brainstem.

We found that BAT of HFD mice administered BARD remained metabolically active, demonstrated by the reduced lipid area and the increase in number of small lipid droplets compared to HFD mice without BARD supplementation. Studies have shown that triterpenes suppress HFD-induced fat deposition in BAT; for instance, the co-administration of ursolic acid increases brown fat in interscapular fat pads of HFD fed mice [25]. Additionally, the CDDO analogues (CDDO-ethyl amide and CDDO-trifluoroethyl amide) eliminate vascuolation in the BAT in a mouse model of Huntington’s disease [44]. BARD has been reported to act as a potent antioxidant inflammatory modulator [45,46,47,48] and body fat suppressor [36,49]. BARD suppresses fat deposition in the visceral fat and liver of diet-induced diabetic mice and HFD-fed mice [16,31]. Furthermore, BARD and its analog RTA 405 reduce body fat leading to reduction of body weight in both animal and human studies [34,36,38,50]. In this study, we have consistently reported the reducing effect of BARD on body weight in HFD group [16]. The consistent increase of body weight and fat deposition in brown fat and many other tissues has been observed in high-fat diet fed mice [51,52,53,54]. Thus, the suprressing effect of BARD on fat deposition in present study demonstrated the potential function of BARD in preventing obesity. Our results are the first to show that BARD prevents fat deposition in the BAT of mice fed a HFD. The reduced fat deposition in BAT of our mouse model is consistent with the pharmacology of BARD. From the outcome of this study, it would be interesting to investigate the effect of BARD on lipid metabolic pathways and lipid metabolism due to their critical involvement in fat deposition and obesity-associated disorders [55,56,57]. 

During obesity there is inflammation of adipose tissue, characterized by the infiltration of macrophages [3,58,59]. The BAT of obesity and diabetic prone mice has elevated levels of macrophages with an inflammatory phenotype [3,24]. In the present study, fewer infiltrating macrophages in the BAT of mice treated with BARD suggest that this triterpenoid compound can prevent HFD-induced inflammation in BAT. BARD suppressed the infiltration of alveolar macrophages in the lung tissue of C57BL/6J mice with bleomycin-induced pulmonary fibrosis [37]. The related triterpene oleanolic acid reduces the infiltration of macrophages and monocytes in the heart tissue of mice with autoimmune myocarditis [60]. Studies have shown that dietary supplementation with pentacyclic triterpenes mediates immune cells (macrophages) and inflammatory cytokines [61,62,63]. This study also supports previous reports on the anti-inflammatory mechanism of BARD [30,64,65,66].

We further found that HFD mice have an increased number of pro-inflammatory and fewer anti-inflammatory macrophages in the BAT, which was inverted by treatment with BARD. Mice in which CD11c expressing cells have been deleted in adipose tissue have increased insulin sensitivity, reduced local and systemic inflammation (decreased level of pro-inflammatory cytokines) and resistance to obesity [67]. Additionally, alternatively activated M2 macrophages in BAT produce catecholamine and are required for adaptive thermogenesis in response to cold [68]. The activation of the M2 macrophage phenotype induces anti-obesity effects through stimulating thermogenesis and insulin sensitivity [68,69]. It has been found that CDDO analogues increase oxygen consumption and induce an anti-inflammatory effect, leading to enhanced insulin sensitivity, and the prevention of inflammation and diabetes in db/db mice [31,70,71]. BARD prevented macrophage infiltration and induced a shift in phenotype from M1 to M2 macrophages, which likely contributes to its anti-obesity effects.

Low sympathetic activity is a common feature of obesity [72]. We found increased TH signalling activity in BAT induced by BARD without changes in UCP1 protein and other thermogenic proteins (β_3_-AR and PGC-1α). It has been shown that sympathetic innervation and cold exposure can activate BAT and directly increase energy expenditure independently of UCP1 [73]. Further, during cold acclimation in mice, BAT is activated and there is an increase in TH-immunoreactivity and the number of brown adipocytes [74]. Data from the present study and from previous reports, showing that BARD treatment increases oxygen consumption in HFD-induced diabetic mice [31], suggest BARD increases energy expenditure. The data further suggest a potential interaction of BARD with the sympathetic nervous system resulting in changes in enegy balance.

The brainstem controls energy balance, and maintains homeostatic functions; and HFD depletes vago-vagal reflex signalling leading to development of obesity [75]. We observed that HFD induced a reduction of TH signalling activity in brainstem VLM that was restored by BARD. Our data suggest that the VLM of the brainstem may be a site of action for BARD, since no effects were observed in the DVC of the brainstem. The increased TH phosphorylation observed in the BAT and WAT of HFD fed mice [16] suggests that oral BARD activates the sympathetic nervous system. These results provide evidence for BARD regulating energy balance in the central nervous system. In addition to the phosphorylation of rate-limiting enzyme (TH) in both BAT and the brainstem by BARD, we further found that this compound increased expression of β_3_-AR and PGC-1α in the VLM of the brainstem of HFD mice. The brainstem contains mitochondrial proteins, which promote oxygen consumption, leading to heat production in the brain [13,76]. It has been previously shown that adrenergic neurons are involved in energy metabolism of the brainstem [77]. PGC-1α is involved in mitochondrial synthesis in the brain [11], and in one report CDDO-methyl amide triterpene increased PGC-1α gene expression in mouse brains [78]. This suggests that BARD mediates energy regulation through the VLM of the brainstem, possibly through adrenergic activation (β_3_-AR) and mitochondrial biogenesis (PGC-1α). The present data and our previous report [16] are an indicator for the involvement of BARD in energy regulation that has similarly mentioned for pentacyclin triterpenes such as ursolic acid, which induces mitochondrial uncoupling and energy expenditure in skeletal muscle of HFD-fed mice [79].

We have shown that oral BARD administration during HFD feeding for 21 weeks in mice prevented fat deposition in the BAT, demonstrated by the reduced size of lipid droplets, and the increased number of small lipid droplets. BARD prevented the development of inflammation in BAT by suppressing the infiltration of macrophages and recruitment of the pro-inflammatory macrophge phenotype. It enhanced noradrenergic activation in BAT and the VLM of the brainstem, assessed by the increased level of TH signalling activity. BARD also enhanced the expression of energy expenditure proteins in the VLM of the brainstem, suggesting potent effects on energy regulation in the brainstem. Additionally, we did not observe any adverse effect under BARD treatment as per the previous reports on BARD and its analogs [80,81]; however, further studies on the toxicity of BARD are worthy for future applications of this compound in the treatment of obesity and associated complications via dietary intervention. Overall, this study is additional evidence for the potential application of BARD in obesity prevention via targeting BAT and the brainstem. 
